# Alteration in behavior of rat after chronic exposure to acetamiprid

**DOI:** 10.14202/vetworld.2019.254-257

**Published:** 2019-02-13

**Authors:** Samiran Mondal, Saktipada Pradhan, Sunit K. Mukhopadhayay

**Affiliations:** Department of Veterinary Pathology, West Bengal University of Animal and Fishery Sciences, Kolkata, West Bengal, India

**Keywords:** acetamiprid, formalin, pain, tail flick

## Abstract

**Background and Aim:**

Acetamiprid is a chemical of neonicotinoid group which binds with nicotinic acetylcholine receptor (nAChR) and alters the brain function. The present study was taken up to enlight the understanding of nociception behavior in Sprague Dawley (SD) rat after multiple exposures to acetamiprid.

**Materials and Methods:**

For experiment purpose, a total of 48 SD rats were divided into four dose groups having 12 animals each. Group I was control group received only distilled water. Group II, Group III, and Group IV were treated with acetamiprid at a dose rate of 5, 20, and 40 mg/kg body weight, respectively. Rats were tested in induced pain by formalin injection and tail flick test.

**Results:**

The flinch counts in formalin-induced pain in acetamiprid-treated rat were reduced in a dose-dependent manner, whereas, in tail flick test, no such altered pain behavior was observed in treated group compared to control animals.

**Conclusion:**

Acetamiprid alters the centralized nociception through nAChR but could not trigger the associated signal to inhibit the nociception peripherally.

## Introduction

As insecticides are used as crop protectant [1], they are likely to cause indirect exposure in human, domestic as well as wild animal and poultry through feed contaminants, soil, and groundwater (in very low amount), as through ecotoxicant [2].

Acetamiprid is an insecticide of neonicotinoid group. It acts through binding with nicotinic acetylcholine receptor (nAChR) in the insect [3]. However, this insecticide has also non-target species effect where it could bind with nAchR present in mammal’s brain [4]. Exposures to acetamiprid cause alteration in brain function in relation to learning and memory [5]. The principal concern is not toxicity resulting from a single or a few large doses of a given pesticide, but due to oral intake of very small quantities over a reasonable period of time. The behavioral changes to these multiple exposures to acetamiprid cause various stress itself in an organism.

Now the question is had this insecticide impairs the learning process, whether a particular stimulus is enough for response of an animal or it lower down the perception in terms of nociception. A particular stimulus for normal unexposed animal would elicit a certain type of behavior as in case of formalin-induced pain model. The present study was designed to understand the fact after multiple exposures to acetamiprid in nociception behavior in Sprague Dawley (SD) rat.

## Materials and Methods

### Ethical approval

Before starting of this experiment, the protocol was approved by the Institutional Animal Ethics Committee (IAEC, constituted under the guidelines of the Committee for the Purpose of Control and Supervision of Experiments on Animals) vide no PHM/583/12. The care and use of laboratory animals were strictly in accordance with the guidelines prescribed by IAEC, WBUAFS.

### Animal experimentation

The present study was conducted on 6-week-old healthy SD rats. The rats were procured and housed in polycarbonate cages (Tarsons) in open cage system at laboratory, Department of Veterinary Pathology, West Bengal University of Animal and Fishery Sciences, W.B. Sterilized husk was used as bedding materials. Changing frequency of bedding materials was twice a week in the evening. The rats were provided *ad libitum* standard feed (Nutrilab, Tetragon Chemie Pvt Ltd, India) and water. Animals were acclimatized in the laboratory for 7 days.

Acetamiprid (Cat# 33674) technical grade was procured from Sigma-Aldrich, USA. Acetamiprid was formulated using distilled water as a vehicle. The dose volume was 10 ml/kg. According to the different dose groups, different concentrations with the same dose volume were freshly prepared each day during the whole study period.

For experiment purpose, a total of 48 animals were used in four dose groups having 12 animals each. Group I was control group received only distilled water. Group II, Group III, and Group IV were treated with acetamiprid at a dose rate of 5, 20, and 40 mg/kg body weight, respectively.

### Formalin-induced pain

This test assessed the centralized pain behavior in rat in a biphasic manner.

#### Habituation

Animals were habituated to the test table by simply keeping the animal for 30 min in two sessions in the 13^th^ week of exposure of acetamiprid. Rats were kept inside a glass box.

#### Test

On the test day, 2.5% formalin was prepared from the stock (Fisher Scientific Chemicals) using distilled water. 50 µl of 2.5% freshly prepared formalin was injected subcutaneously in the right hind paw of each animal. Injection was given in such a way that, after pricking, the tip of needle would not touch again the skin upper neath. Direction of needle was from phalanges toward heel. Insulin syringe (BD) with 31-gauge needle was used for injection. Soon after injection, animal was kept inside the glass box for observation. Only flinches were counted manually for 1 h duration to read the pain behavior in this experiment. The readout was presented in 5 min bin time.

### Tail flick test

For tail flick test, instrument from UGO Basile was used. Animals were habituated with the instrument and handling technique 1 day before the experiment. The infrared beam was given 6 cm from the tip of the tail. Latency to remove the tail from the beam was measured. For the experiment, 60 IR was set with 20 s ramp time. Tail flick test was conducted at the 13^th^ week of exposure.

## Results

### Formalin-induced pain behavior

**Figure-1:**
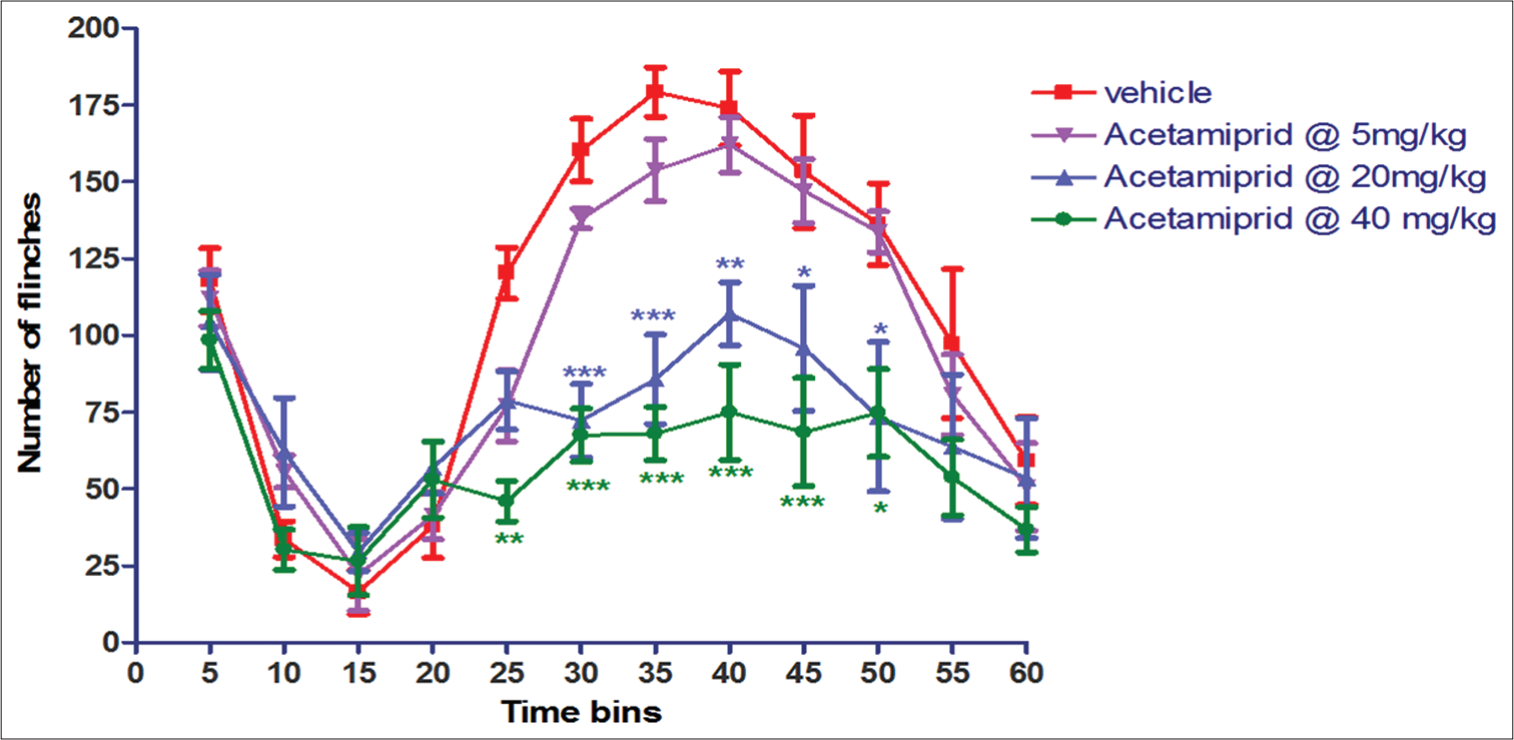
Effect of acetamiprid on formalin-induced pain behavior in Sprague Dawley rat.

Data are depicted in Figure-1. There was a clear dose response in pain sensation induced by injection of formalin. Response of acetamiprid-exposed animal showed less pain behavior in terms of flinching. There was no difference in flinching number in Group II in any time bins compared to vehicle. On the other hand, rats of Group III showed significant (p≤0.05) decrease in flinch count in the time bins of 30, 35, 40, 45, and 50 min compared to rats of Group I. Similarly, in Group IV, the number of flinches was reduced significantly (p≤0.05) in the time bins of 25, 30, 35, 40, 45, and 50 min during testing in formalin-induced pain behavior in SD rat.

### Tail flick test

**Figure-2:**
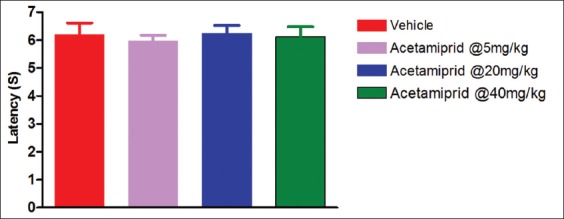
Effect of acetamiprid in tail flick test.

Data are presented in Figure-2. There was no significant difference between exposed animal and control animal in the present experiment.

## Discussion

The antinociceptive response may be due to inhibitory synaptic transmission enhancement in the dorsal horn of the spinal cord through activation of α4β2 nAChRs on pathological pain models. In the present experiment, 13-week acetamiprid-treated rats showed antinociception in formalin-induced pain in SD rats. There was activation of α4β2 subtype of nAChRs in mice by selective agonist usage in formalin-induced pain [6]. The mechanism underlying formalin-induced pain behavior involves a complex series of events including peripheral and central biphasic responses. The first phase of the response is driven directly by formalin stimulating to peripheral nociceptors, thereby producing an acute barrage of activity in the dorsal horn. The second phase is thought to be the consequence of ongoing afferent input maintained by inflammatory mediators acting on peripheral nociceptors [7] and functional changes in central pain processing. A high-affinity ligand for nAChR can conduct Ca2+ ions and thereby directly impact neurotransmitter release [7]. As acetamiprid is also an agonist to nAChR, it might inhibit elicitation of pain response in SD rat.

The nAChR agonists display a wide-range profile of antinociceptive activity in acute, tonic, and chronic pain models. Compounds that act at nAChRs in the central nervous system (CNS) and periphery have been reported to show antinociceptive activity in several rodent acute and chronic pain models [8]. nAChRs are ion channels which are ligand-gated and subunits (α and β subunits) assembled to form homo or heteropentomers [9], which are widely distributed in the peripheral and CNS. These nAChRs are expressed in many areas of CNS, including midbrain [10], medulla [11], nucleus raphe magnus [12], thalamus, pedunculopontine tegmental nucleus [13], and spinal cord [14] to contribute in pain sensation. These nAChR subunits are found in both CNS nucleus and spinal cord [15]. Although α3β4 is the major subtype expressed in peripheral ganglia [16], it is also expressed in the medial habenula, cerebellum, and spinal cord [17]. Over the past several years, α4β2 nicotinic agonists were reported to display a wide-range profile of antinociceptive activity in acute models (such as tail flick and hot plate tests), tonic or persistent models (such as the formalin test), and chronic pain models [18]. AlSharari *et al*. [19] pointed out in acute thermal and tonic pain mouse models that the antinociceptive effects of varenicline and sazetidine A, two new α4β2 nicotinic partial agonists, failed to induce significant effects in the tail flick and hot plate tests after subcutaneous administration. In the present study, acetamiprid also did not produce any effect in tail flick model. It may due to the complex mechanism involved in tail flick test. Although acetamiprid acts as an agonist to nAChR, it could not trigger the associated signal to inhibit the nociception.

## Conclusion

Acetamiprid acts as a ligand to the nAChR and conducts calcium ions to inhibit the release of neurotransmitter even after the presence of inflammatory mediators acting on peripheral nociceptors which cause functional changes in central pain processing.

## Authors’ Contributions

All authors participated equally in the experimental plan and design. SM carried out the whole study. SP processed samples and SKM supervised the whole experiment. All authors read and approved the final manuscript.
